# Density functional theory calculated data of the iodomethane oxidative addition to oligothiophene-containing rhodium complexes – Importance of dispersion correction

**DOI:** 10.1016/j.dib.2021.106929

**Published:** 2021-03-03

**Authors:** Nandisiwe Ghandi Sibongile Mateyise, Jeanet Conradie, Marrigje M. Conradie

**Affiliations:** Department of Chemistry, University of the Free State, PO Box 339, 9300 Bloemfontein, South Africa

**Keywords:** Rhodium, Oxidative addition, DFT, Oligothiophene

## Abstract

Electronic and free energy data of density functional theory calculated optimized geometries of the reactants, transition state of the oxidative addition reaction and different reaction products of the [Rh(RCOCHCOCF_3_)(CO)(PPh_3_)] + CH_3_I reactions (R = C_4_H_3_S, C_4_H_3_S-C_4_H_2_S and C_4_H_3_S-C_4_H_2_S-C_4_H_2_S) are presented to illustrate the influence of the amount of thiophene groups, the implicit solvent and dispersion correction on the calculated energies. All calculations were done with the B3LYP functional, in gas as well as in solvent phase, with and without dispersion correction. The data can save computational chemists time when choosing an appropriate method to calculate reaction energies of oxidative addition reactions. Detailed knowledge of energies involved in the oxidative addition reaction of methyl iodide to rhodium complexes have an important implication in catalysis, for example the Monsanto process where methanol is converted to acetic acid catalysed by a rhodium complex. For more insight in the reported data, see the related research article “Synthesis, characterization, electrochemistry, DFT and kinetic study of the oligothiophene-containing complex [Rh((C_4_H_3_S-C_4_H_2_S)COCHCOCF_3_)(CO)(PPh_3_)]”, published in Polyhedron [1].

## Specifications Table

**Table:** 

Subject	Physical and Theoretical Chemistry
Specific subject area	DFT calculations of chemical structures.
Type of data	Table
	Graph
	Figure
How data were acquired	Electronic structure calculations, using the Gaussian 16 program
Data format	Raw and Analyzed
Parameters for data collection	Geometry optimization and frequency calculations were done using the Gaussian 16 program, with and without the implicit solvent model IEF-PCM, using the B3LYP functional with and without D3 dispersion correction.
Description of data collection	Data were collected from DFT output files
Data source location	University of the Free State
	Bloemfontein
	South Africa
Data accessibility	With the article
Related research article	N G.S. Mateyise, M.M Conradie, Jeanet Conradie, Synthesis, Characterization, Electrochemistry, DFT and Kinetic Study of the Oligothiophene-containing Complex [Rh((C_4_H_3_S-C_4_H_2_S)COCHCOCF_3_)(CO)(PPh_3_)], Polyhedron, 115,095 (2021), https://doi.org/10.1016/j.poly.2021.115095

## Value of the Data

• Free energy data involved in oxidative addition reactions are important in the field of catalysis such as the oxidative addition reaction involved in the manufacturing of methanol from acetic acid (Monsanto process).
• Free energy data obtained by different computational chemistry approaches, namely in gas and solvent phase, with and without dispersion corrections helps computational chemistry researchers in the choice of method when calculating energies involved in oxidative addition reactions.
• Free energy data obtained by different computational chemistry approaches, indicates which method gives energies in agreement with experiment, making the theoretical prediction of energies involved in related oxidation addition reactions possible.

## 1 Data Description

**Scheme 1:**
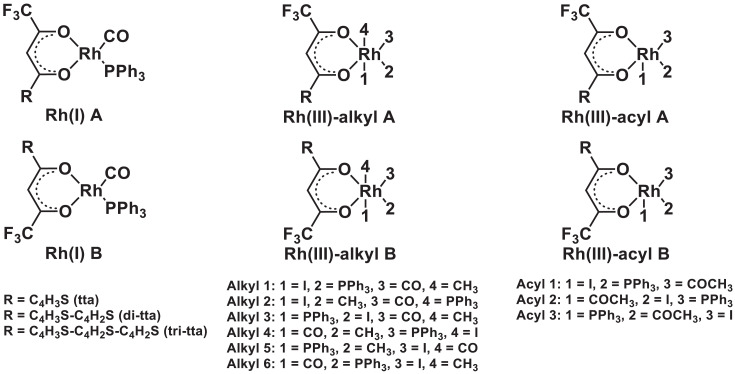
Rhodium(I) and (III) complexes of this study. For each Rh(I), Rh(III)-alkyl and Rh(III)-acyl, two geometrical isomers are possible, namely A and B.

**Fig. 1:**
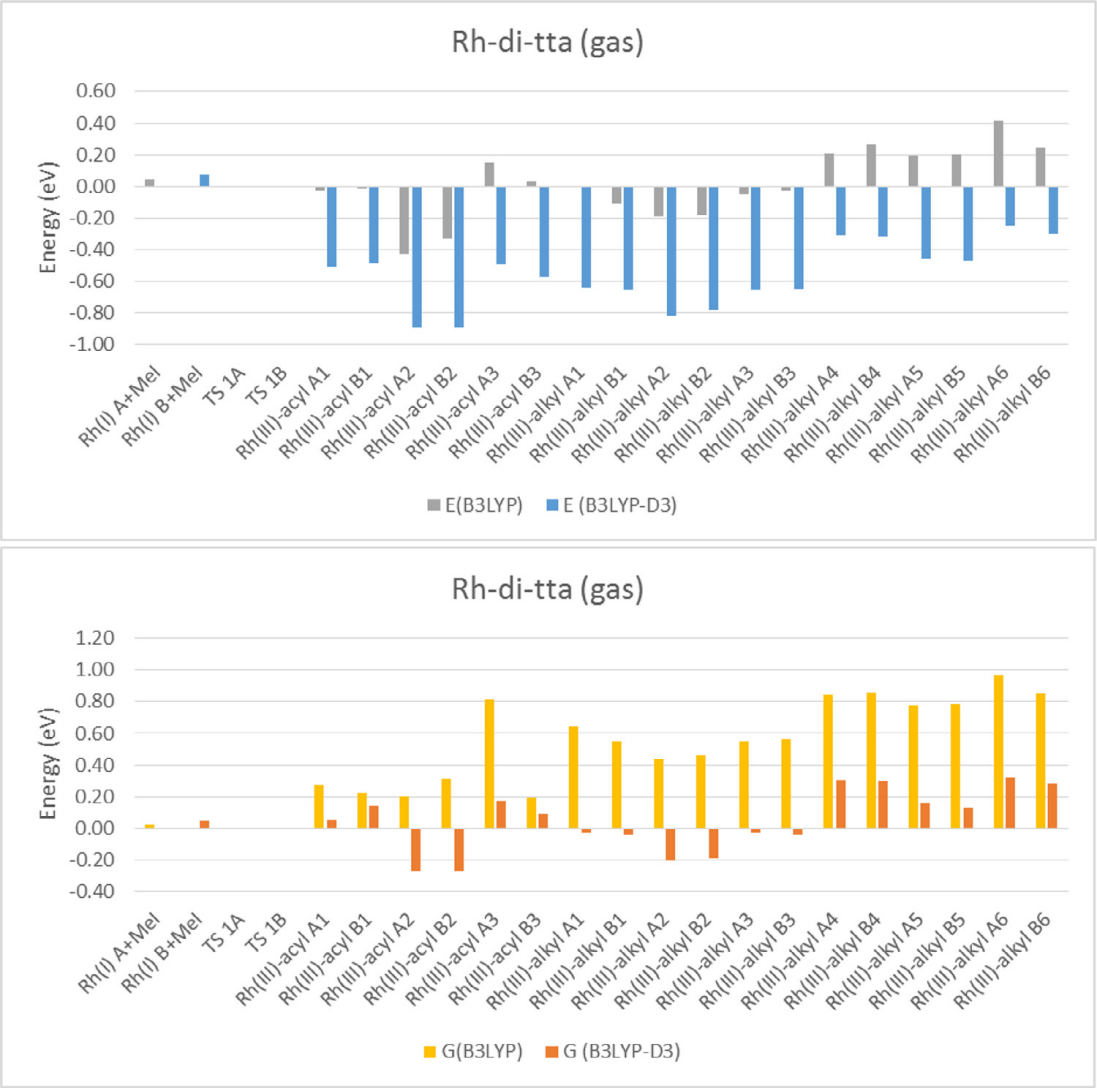
B3LYP and B3LYP-D3 gas phase relative electronic (E) and free (G) energies of Rh(III)-di-tta complexes compared to the lowest energy reactant isomer Rh(I) + CH_3_I (MeI), illustrating the influence of the dispersion correction on the gas phase calculated energy. No oxidative addition TS could be located in the gas phase.

**Fig. 2:**
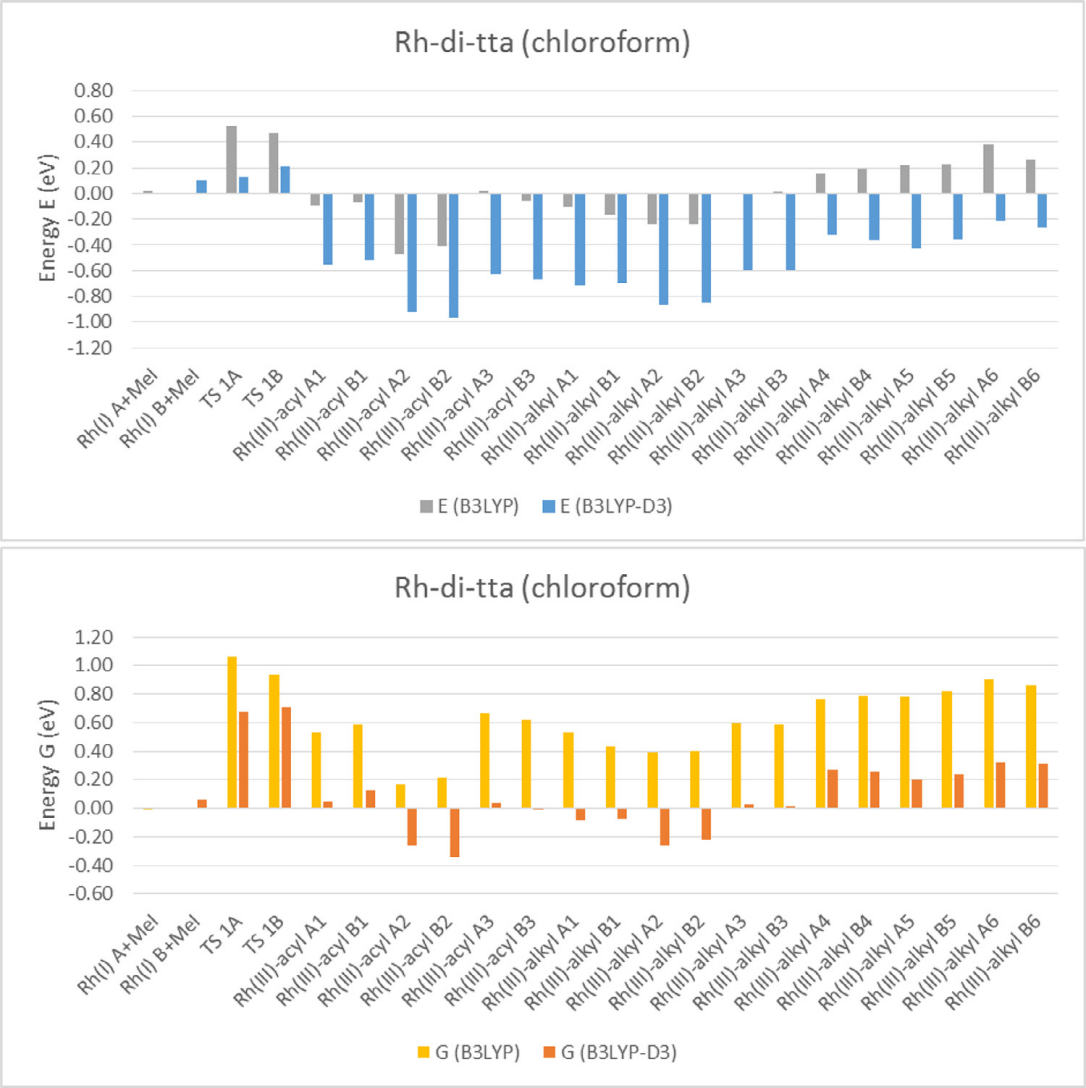
B3LYP and B3LYP-D3 solvent (chloroform) phase relative electronic (E) and free (G) energies of Rh(III)-di-tta complexes compared to the lowest energy reactant isomer Rh(I) + CH_3_I (MeI), illustrating the influence of the dispersion correction on the solvent (chloroform) phase calculated energy.

**Fig. 3:**
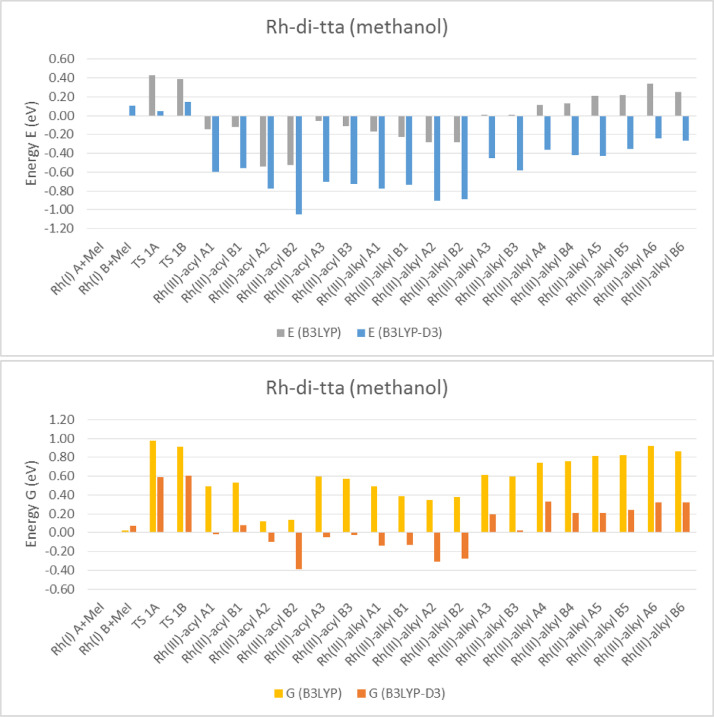
B3LYP and B3LYP-D3 solvent (methanol) phase relative electronic (E) and free (G) energies of Rh(III)-di-tta complexes compared to the lowest energy reactant isomer Rh(I) + CH_3_I (MeI), illustrating the influence of the dispersion correction on the solvent (methanol) phase calculated energy.

**Fig. 4:**
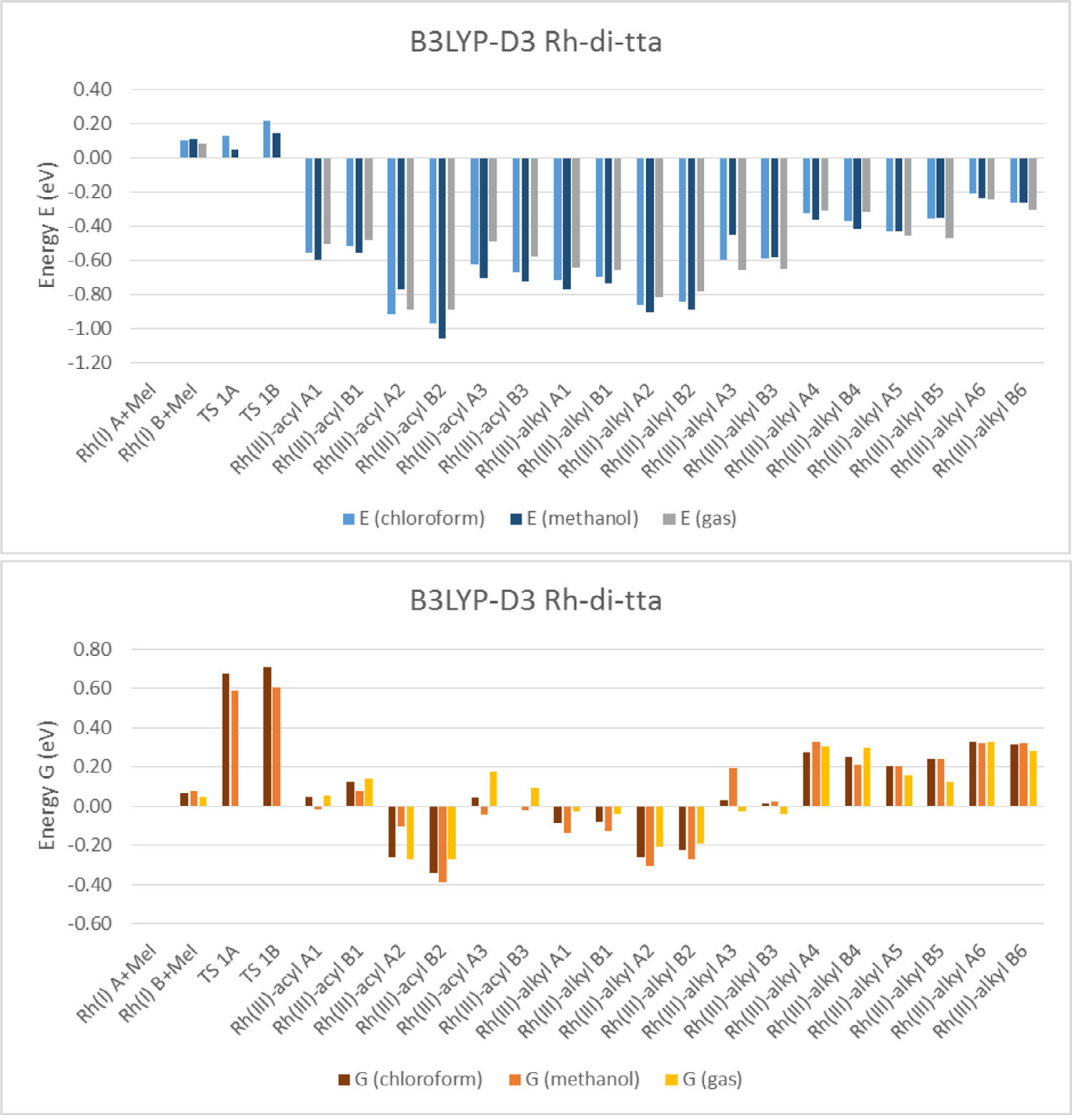
B3LYP-D3 relative electronic (E) and free (G) energies of Rh(III)-di-tta complexes compared to the lowest energy reactant isomer Rh(I) + CH_3_I (MeI), illustrating the influence of the phase (gas, chloroform or methanol) on the calculated energy. No oxidative addition TS could be located in the gas phase.

**Fig. 5:**
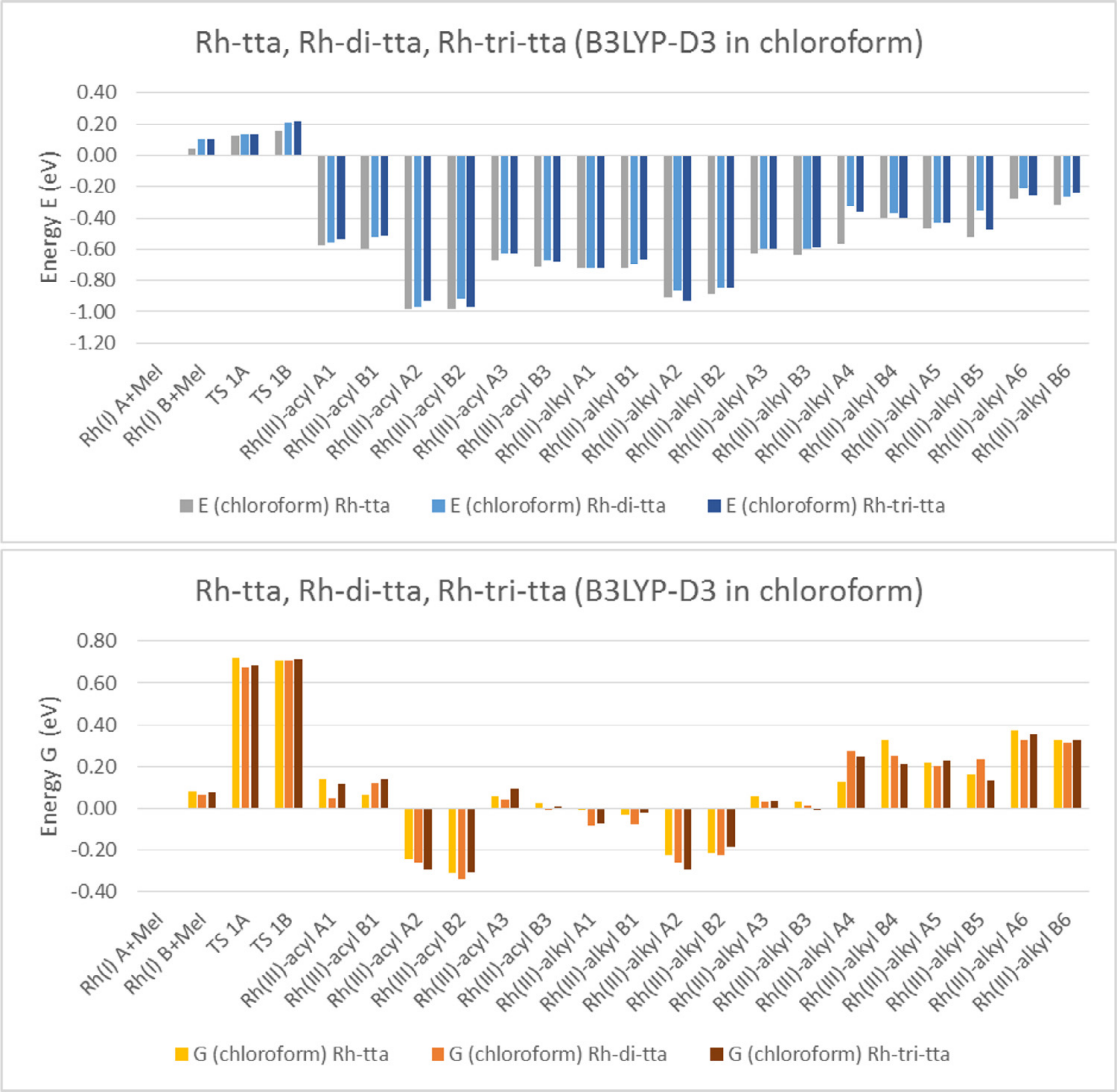
B3LYP-D3 solvent (chloroform) phase relative electronic (E) and free (G) energies of Rh(III)-tta, Rh(III)-di-tta and Rh(III)-tri-tta complexes compared to the lowest energy reactant isomer Rh(I) + Rh(I) + CH_3_I (MeI), illustrating the influence of the amount of thienyl groups on the calculated energy.

Electronic and free energy data of the reactants, first transition state (TS) and the possible reaction products of [Rh(RCOCHCOCF_3_)(CO)(PPh_3_)] + CH_3_I reaction (R = C_4_H_3_S (tta) [2], C_4_H_3_S-C_4_H_2_S (di-tta) [1] and C_4_H_3_S-C_4_H_2_S-C_4_H_2_S (tri-tta)) shown in Scheme 1, are specified in the graphs in Figs. 1–5. The influence of dispersion correction to the energy data of the Rh(I)-di-tta + CH_3_I reaction (R = C_4_H_3_S-C_4_H_2_S) is illustrated in Fig. 1 (gas phase data), Fig. 2 (data in chloroform as solvent) and Fig. 3 (data in methanol as solvent). The influence of the phase (gas, chloroform or methanol) to the energy data of the Rh(I)-di-tta + CH_3_I reaction (R = C_4_H_3_S-C_4_H_2_S) is illustrated in Fig. 4 (B3LYP-D3 data). The influence of the amount of thienyl groups to the energy data of the Rh(I) + CH_3_I reaction (R = C_4_H_3_S (tta), C_4_H_3_S-C_4_H_2_S (di-tta) and C_4_H_3_S-C_4_H_2_S-C_4_H_2_S (tri-tta)) is illustrated in Fig. 5 (B3LYP-D3 data in chloroform as solvent). The energies of the products, relative to the energy of the reactants, show if a reaction product is thermodynamically favoured. The electronic and free energy data presented in Figs. 1–5 are provided in Table 1. The B3LYP-D3 data in chloroform as solvent of [Rh((C_4_H_3_S-C_4_H_2_S)COCHCOCF_3_)(CO)(PPh_3_)] + CH_3_I is from the related research article [1]. Experimental and theoretical data of reaction involving the mother complex, [Rh(CH_3_COCHCOCH_3_)(CO)(PPh_3_)], and related complexes, [Rh(RCOCHCOC_4_H_3_S)(CO)(PPh_3_)] (R = C_6_H_5_ and C_4_H_3_333\S), can be found in references [3], [4], [5].

## 2 Experimental Design, Materials and Methods

Density functional theory (DFT) calculations using the Gaussian 16 package [6], were used to determine the optimized geometry and energy of the spesified molecules. The input coordinates for the compounds were constructed using Chemcraft [7]. The coordinates were spesified in the input files of the DFT calculations. DFT calculations were performed using the hybrid functional B3LYP functional [8], [9] applying the GTO (Gaussian type orbital) triple-ζ basis set 6–311G(d,p) for the lighter atoms (C, H, O, F) and the Lanl2dz basis set [10], that corresponds to the Los Alamos ECP plus DZ, for Rh and I. The optimization is performed using Berny algorithm using GEDIIS [11] as implemented in Gaussian 16. The convergence is reached when the root mean square force, the maximum force, the root mean square displacement and the maximum displacement are within the threshold of 0.00030, 0.00045, 0.0012 and 0.0018 atomic units, respectively. The requested convergence on energy is 1.0D-8 atomic unit. Calculations were done with and without Grimme's D3 dispersion correction [12], in gas and solvent phase, using either chloroform or methanol as solvent. For solvent calculations, the integral equation formalism polarizable continuum model (IEFPCM) of solvation to describe the dielectric continuum medium, was used [13,14]. Frequency calculations were done on all molecules to ensure true minimum energy (no imaginary frequency) or transtion state structure (one imaginary frequency), and to provide the free energies of the molcules. The free energies were obtained from the output files searching for “Sum of electronic and thermal Free Energies=”. The electronic energies were obtained from the output files at the final optimization step, searching for “SCF Done” from the bottom of the output file.

**Table 1:** Electronic (E (eV)) and free energy (G (eV)) data of the indicated reaction products of the [Rh(RCOCHCOCF_3_)(CO)(PPh_3_)] + CH_3_I (MeI), reaction (R = C_4_H_3_S (tta), C_4_H_3_S-C_4_H_2_S (di-tta) and C_4_H_3_S-C_4_H_2_S-C_4_H_2_S (tri-tta)) calculated with B3LYP (with and without dispersion correction) and the indicated phase (gas, chloroform or methanol).

	Rh-tta		Rh-di-tta												Rh-tri-tta	
	B3LYP-D3		B3LYP		B3LYP-D3										B3LYP-D3	
	CHCl_3_		Gas		CHCl_3_		MeOH		Gas		CHCl_3_		MeOH		CHCl_3_	
	ΔE	ΔG	ΔE	ΔG	ΔE	ΔG	ΔE	ΔG	ΔE	ΔG	ΔE	ΔG	ΔE	ΔG	ΔE	ΔG
Rh(I) A + CH_3_I	0.00	0.00	0.05	0.03	0.02	0.00	0.00	0.00	0.00	0.00	0.00	0.00	0.00	0.00	0.00	0.00
Rh(I) B + CH_3_I	0.04	0.08	0.00	0.00	0.00	0.00	0.00	0.02	0.08	0.05	0.10	0.06	0.11	0.07	0.10	0.08
TS 1A	0.13	0.72	–	–	0.53	1.06	0.43	0.98	–	–	0.13	0.67	0.05	0.59	0.13	0.68
TS 1B	0.16	0.71	–	–	0.47	0.94	0.39	0.91	–	–	0.21	0.71	0.14	0.60	0.21	0.72
Rh(III)-acyl A1	−0.58	0.14	−0.03	0.28	−0.09	0.53	−0.14	0.49	−0.51	0.05	−0.56	0.04	−0.60	−0.02	−0.54	0.11
Rh(III)-acyl B1	−0.60	0.06	−0.01	0.22	−0.07	0.58	−0.12	0.53	−0.48	0.14	−0.52	0.12	−0.55	0.08	−0.51	0.14
Rh(III)-acyl A2	−0.99	−0.24	−0.43	0.20	−0.47	0.16	−0.54	0.12	−0.89	−0.27	−0.92	−0.26	−0.77	−0.10	−0.93	−0.29
Rh(III)-acyl B2	−0.99	−0.31	−0.33	0.31	−0.41	0.21	−0.53	0.14	−0.89	−0.27	−0.97	−0.34	−1.05	−0.39	−0.97	−0.31
Rh(III)-acyl A3	−0.67	0.06	0.15	0.81	0.02	0.67	−0.06	0.60	−0.49	0.17	−0.63	0.04	−0.70	−0.05	−0.63	0.09
Rh(III)-acyl B3	−0.71	0.02	0.03	0.20	−0.06	0.62	−0.12	0.57	−0.58	0.09	−0.67	−0.01	−0.72	−0.02	−0.68	0.01
Rh(III)-alkyl A1	−0.72	−0.01	0.00	0.64	−0.10	0.53	−0.17	0.49	−0.64	−0.03	−0.72	−0.09	−0.77	−0.14	−0.72	−0.08
Rh(III)-alkyl B1	−0.72	−0.03	−0.11	0.55	−0.17	0.44	−0.22	0.39	−0.66	−0.04	−0.70	−0.08	−0.74	−0.13	−0.66	−0.02
Rh(III)-alkyl A2	−0.91	−0.23	−0.19	0.44	−0.24	0.39	−0.28	0.35	−0.81	−0.21	−0.86	−0.26	−0.90	−0.31	−0.93	−0.29
Rh(III)-alkyl B2	−0.89	−0.22	−0.18	0.46	−0.24	0.40	−0.28	0.37	−0.78	−0.19	−0.85	−0.23	−0.89	−0.27	−0.85	−0.19
Rh(III)-alkyl A3	−0.63	0.06	−0.05	0.55	0.00	0.60	0.01	0.61	−0.66	−0.03	−0.60	0.03	−0.45	0.19	−0.60	0.04
Rh(III)-alkyl B3	−0.63	0.03	−0.03	0.56	0.01	0.58	0.01	0.59	−0.65	−0.04	−0.59	0.01	−0.58	0.02	−0.59	−0.01
Rh(III)-alkyl A4	−0.56	0.13	0.21	0.84	0.16	0.77	0.11	0.74	−0.31	0.30	−0.32	0.27	−0.36	0.33	−0.36	0.25
Rh(III)-alkyl B4	−0.40	0.33	0.26	0.86	0.19	0.79	0.13	0.76	−0.32	0.30	−0.37	0.25	−0.42	0.21	−0.40	0.21
Rh(III)-alkyl A5	−0.47	0.22	0.19	0.78	0.22	0.78	0.21	0.81	−0.46	0.16	−0.43	0.20	−0.43	0.20	−0.43	0.23
Rh(III)-alkyl B5	−0.52	0.16	0.20	0.78	0.22	0.82	0.22	0.82	−0.47	0.12	−0.36	0.24	−0.35	0.24	−0.48	0.13
Rh(III)-alkyl A6	−0.28	0.37	0.41	0.97	0.38	0.90	0.34	0.92	−0.25	0.32	−0.21	0.32	−0.24	0.32	−0.26	0.36
Rh(III)-alkyl B6	−0.32	0.33	0.25	0.85	0.26	0.86	0.25	0.87	−0.30	0.28	−0.27	0.31	−0.26	0.32	−0.24	0.33

## Ethics Statement

This work does not require any ethical statement.

## CRediT Author Statement

**Nandisiwe Ghandi Sibongile Mateyise:** DFT calculations, Data curation; **Marrigje M. Conradie:** Conceptualization, Supervision, Methodology, Reviewing and Editing; **Jeanet Conradie:** Supervision, Methodology, DFT calculations, Data curation, Writing - Reviewing and Editing.

## Declaration of Competing Interest

The authors declare that they have no known competing financial interests or personal relationships which have or could be perceived to have influenced the work reported in this article.

## Appendix Supplementary materials

Image, application 1
