# Supplementary material for: Density functional theory calculated data of the iodomethane oxidative addition to oligothiophene-containing rhodium complexes – Importance of dispersion correction
Source: Data Brief. 2021 Mar 3;35:106929. doi: 10.1016/j.dib.2021.106929 (PMC7973308; doi:10.1016/j.dib.2021.106929)
Supplement: Supplementary file 1 [file mmc1.docx]

**Density functional theory calculated data of the iodomethane oxidative addition to oligothiophene-containing rhodium complexes – importance of dispersion correction**

**Authors**

Nandisiwe Ghandi Sibongile Mateyise, Jeanet Conradie and Marrigje M Conradie

**Affiliations**

Department of Chemistry, PO Box 339, University of the Free State, 9300 Bloemfontein, Republic of South Africa.

**Corresponding author(s)**

Marrigje M Conradie (ConradieMM@ufs.ac.za)

**Abstract**

Electronic and free energy data of density functional theory calculated optimized geometries of the reactants, transition state of the oxidative addition reaction and different reaction products of the [Rh(RCOCHCOCF_3_)(CO)(PPh_3_)] + CH_3_I reactions (R = C_4_H_3_S, C_4_H_3_S-C_4_H_2_S and C_4_H_3_S-C_4_H_2_S-C_4_H_2_S) are presented to illustrate the influence of the amount of thiophene groups, the implicit solvent and dispersion correction on the calculated energies. All calculations were done with the B3LYP functional, in gas as well as in solvent phase, with and without dispersion correction. The data can save computational chemists time when choosing an appropriate method to calculate reaction energies of oxidative addition reactions. Detailed knowledge of energies involved in the oxidative addition reaction of methyl iodide to rhodium complexes have an important implication in catalysis, for example the Monsanto process where methanol is converted to acetic acid catalysed by a rhodium complex. For more insight in the reported data, see the related research article “Synthesis, characterization, electrochemistry, DFT and kinetic study of the oligothiophene-containing complex [Rh((C_4_H_3_S-C_4_H_2_S)COCHCOCF_3_)(CO)(PPh_3_)]”, published in Polyhedron [1].

**Keywords**

rhodium; oxidative addition; DFT; oligothiophene

**Specifications Table**

| **Subject** | Physical and Theoretical Chemistry |
| --- | --- |
| **Specific subject area** | DFT calculations of chemical structures. |
| **Type of data** | Table  Graph  Figure |
| **How data were acquired** | Electronic structure calculations, using the Gaussian 16 program |
| **Data format** | Raw and Analyzed |
| **Parameters for data collection** | Geometry optimization and frequency calculations were done using the Gaussian 16 program, with and without the implicit solvent model IEF-PCM, using the B3LYP functional with and without D3 dispersion correction. |
| **Description of data collection** | Data were collected from DFT output files |
| **Data source location** | University of the Free State  Bloemfontein  South Africa |
| **Data accessibility** | With the article |
| **Related research article** | N G.S. Mateyise, M.M Conradie, Jeanet Conradie, Synthesis, Characterization, Electrochemistry, DFT and Kinetic Study of the Oligothiophene-containing Complex [Rh((C_4_H_3_S-C_4_H_2_S)COCHCOCF_3_)(CO)(PPh_3_)], Polyhedron, 115095 (2021) DOI 10.1016/j.poly.2021.115095 |

**Value of the Data**

- Free energy data involved in oxidative addition reactions are important in the field of catalysis such as the oxidative addition reaction involved in the manufacturing of methanol from acetic acid (Monsanto process).
- Free energy data obtained by different computational chemistry approaches, namely in gas and solvent phase, with and without dispersion corrections helps computational chemistry researchers in the choice of method when calculating energies involved in oxidative addition reactions.
- Free energy data obtained by different computational chemistry approaches, indicates which method gives energies in agreement with experiment, making the theoretical prediction of energies involved in related oxidation addition reactions possible.

**Data Description**

Electronic and free energy data of the reactants, first transition state (TS) and the possible reaction products of [Rh(RCOCHCOCF_3_)(CO)(PPh_3_)] + CH_3_I reaction (R = C_4_H_3_S (tta) [2], C_4_H_3_S-C_4_H_2_S (di-tta) [1] and C_4_H_3_S-C_4_H_2_S-C_4_H_2_S (tri-tta)) shown in Scheme 1, are specified in the graphs in Figure 1 – Figure 5. The influence of dispersion correction to the energy data of the Rh(I)-di-tta + CH_3_I reaction (R = C_4_H_3_S-C_4_H_2_S) is illustrated in Figure 1 (gas phase data), Figure 2 (data in chloroform as solvent) and Figure 3 (data in methanol as solvent). The influence of the phase (gas, chloroform or methanol) to the energy data of the Rh(I)-di-tta + CH_3_I reaction (R = C_4_H_3_S-C_4_H_2_S) is illustrated in Figure 4 (B3LYP-D3 data). The influence of the amount of thienyl groups to the energy data of the Rh(I) + CH_3_I reaction (R = C_4_H_3_S (tta), C_4_H_3_S-C_4_H_2_S (di-tta) and C_4_H_3_S-C_4_H_2_S-C_4_H_2_S (tri-tta)) is illustrated in Figure 5 (B3LYP-D3 data in chloroform as solvent). The energies of the products, relative to the energy of the reactants, show if a reaction product is thermodynamically favoured. The electronic and free energy data presented in Figure 1 – Figure 5 are provided in Table 1. The B3LYP-D3 data in chloroform as solvent of [Rh((C_4_H_3_S-C_4_H_2_S)COCHCOCF_3_)(CO)(PPh_3_)] + CH_3_I is from the related research article [1]. Experimental and theoretical data of reaction involving the mother complex, [Rh(CH_3_COCHCOCH_3_)(CO)(PPh_3_)], and related complexes, [Rh(RCOCHCOC_4_H_2_S)(CO)(PPh_3_)] (R = C_6_H_5_ and C_4_H_2_S), can be found in references [3–5].

Scheme 1. Rhodium(I) and (III) complexes of this study. For each Rh(I), Rh(III)-alkyl and Rh(III)-acyl, two geometrical isomers are possible, namely A and B.


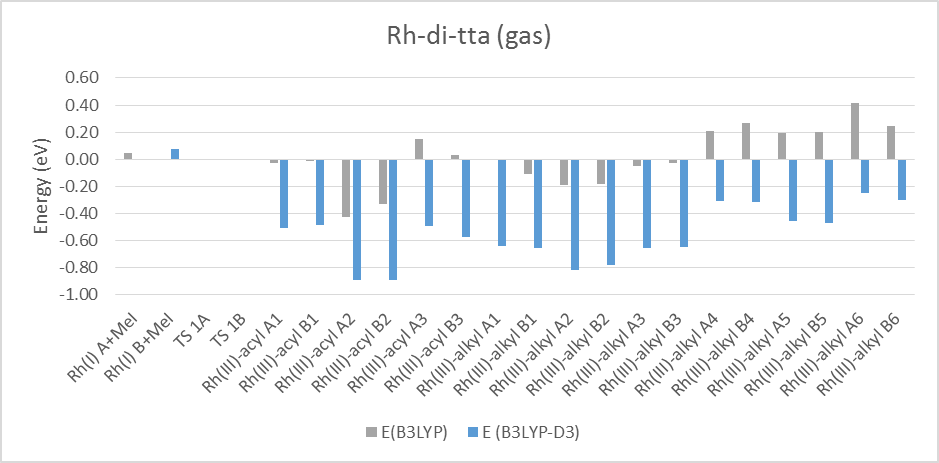


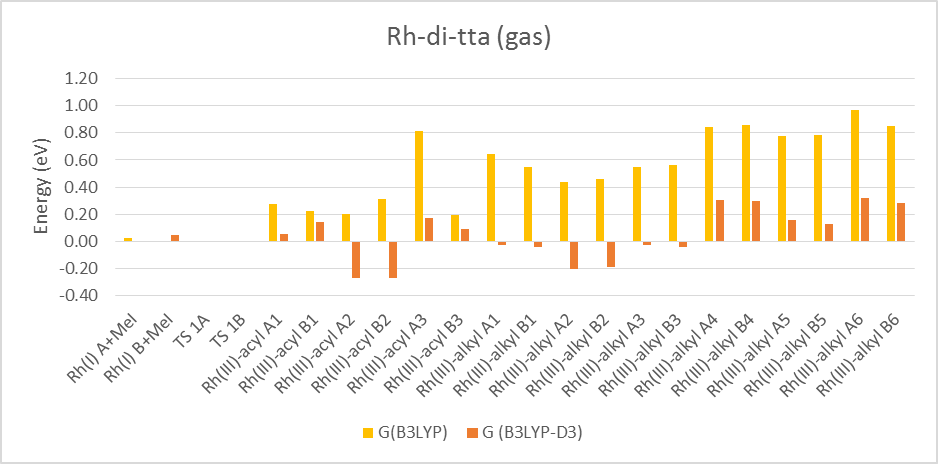


Figure 1. B3LYP and B3LYP-D3 gas phase relative electronic (E) and free (G) energies of Rh(III)-di-tta complexes compared to the lowest energy reactant isomer Rh(I) + CH_3_I (MeI), illustrating the influence of the dispersion correction on the gas phase calculated energy. No oxidative addition TS could be located in the gas phase.


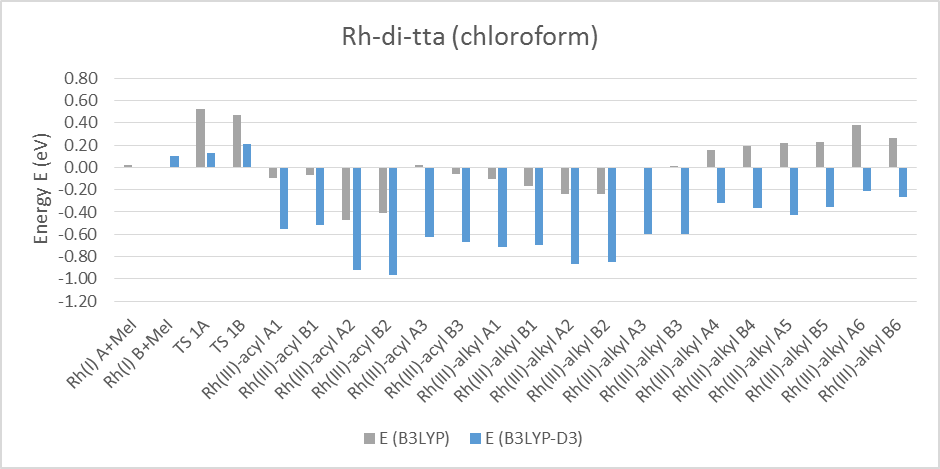

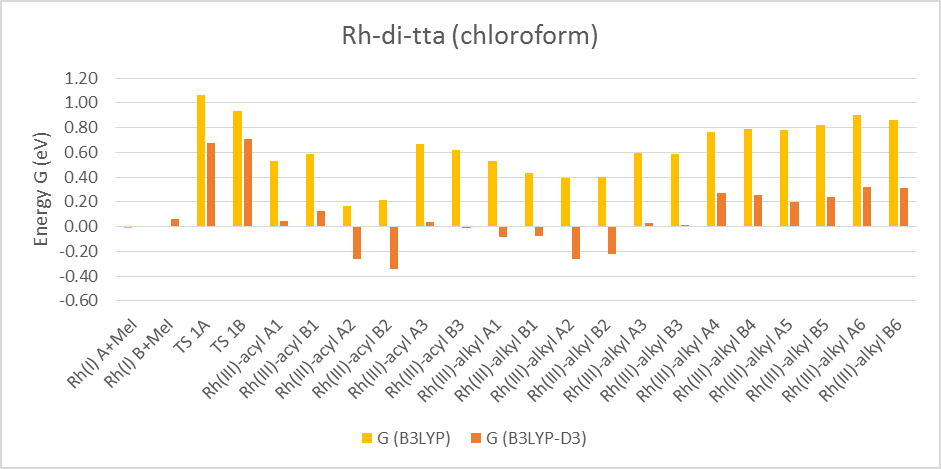


Figure 2. B3LYP and B3LYP-D3 solvent (chloroform) phase relative electronic (E) and free (G) energies of Rh(III)-di-tta complexes compared to the lowest energy reactant isomer Rh(I) + CH_3_I (MeI), illustrating the influence of the dispersion correction on the solvent (chloroform) phase calculated energy.


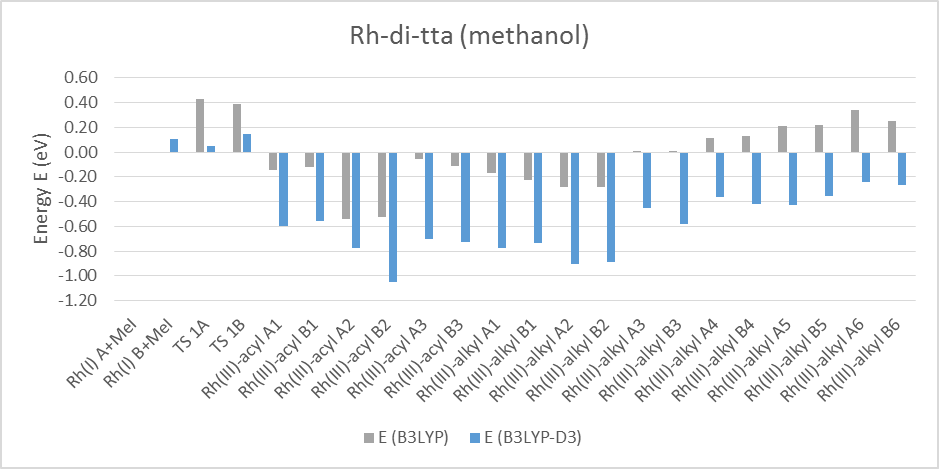

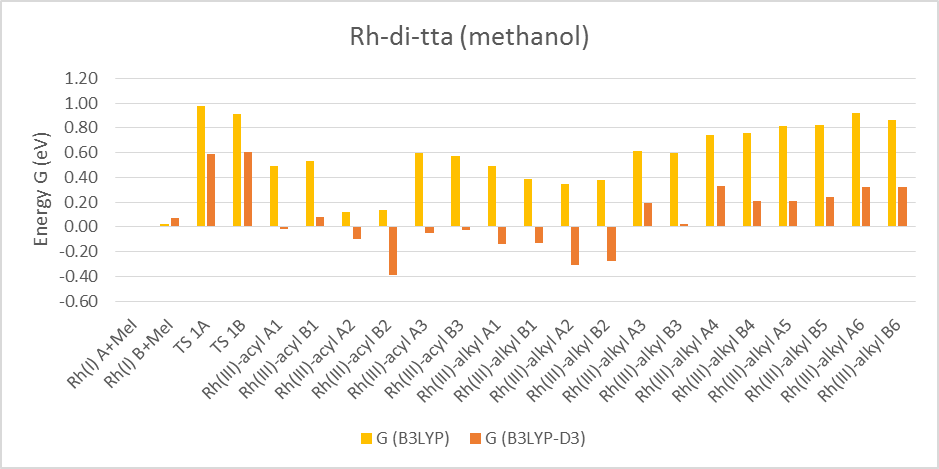


Figure 3. B3LYP and B3LYP-D3 solvent (methanol) phase relative electronic (E) and free (G) energies of Rh(III)-di-tta complexes compared to the lowest energy reactant isomer Rh(I) + CH_3_I (MeI), illustrating the influence of the dispersion correction on the solvent (methanol) phase calculated energy.


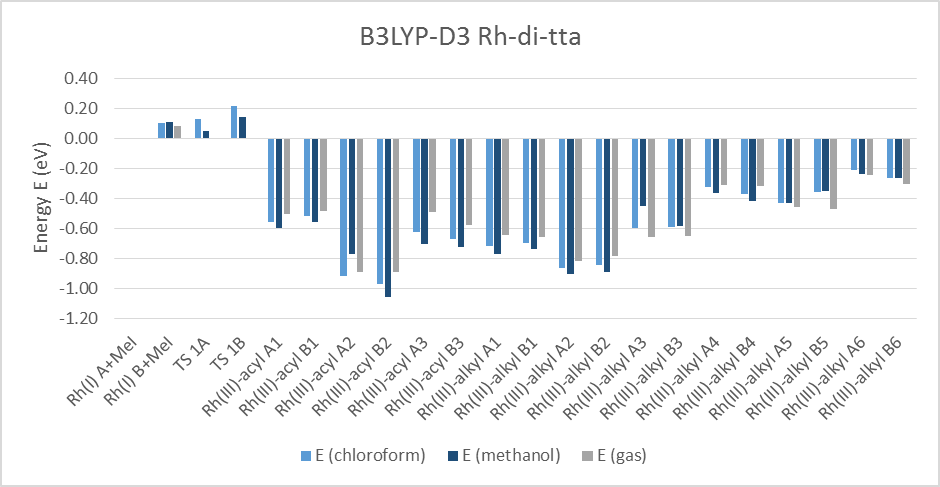

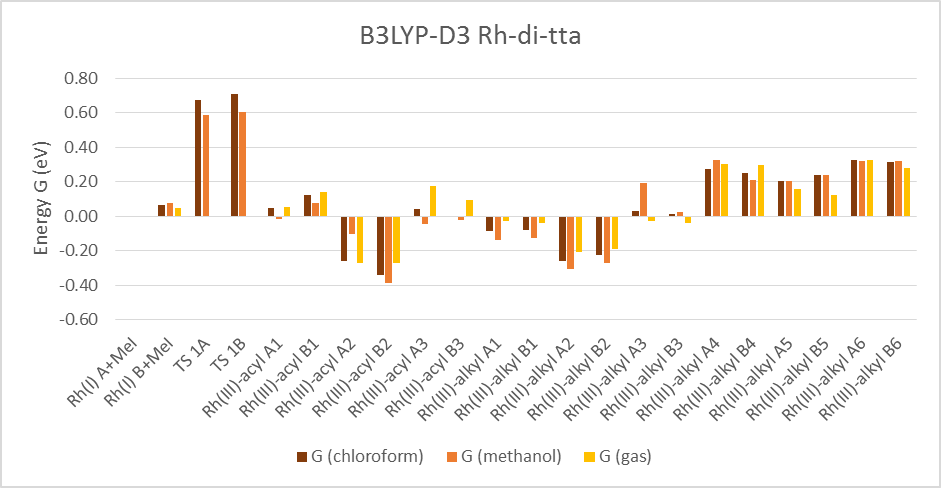


Figure 4. B3LYP-D3 relative electronic (E) and free (G) energies of Rh(III)-di-tta complexes compared to the lowest energy reactant isomer Rh(I) + CH_3_I (MeI), illustrating the influence of the phase (gas, chloroform or methanol) on the calculated energy. No oxidative addition TS could be located in the gas phase.


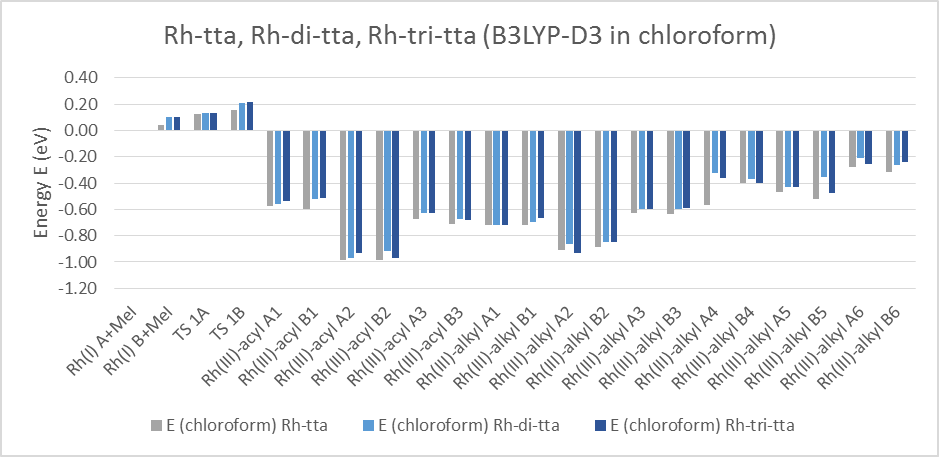


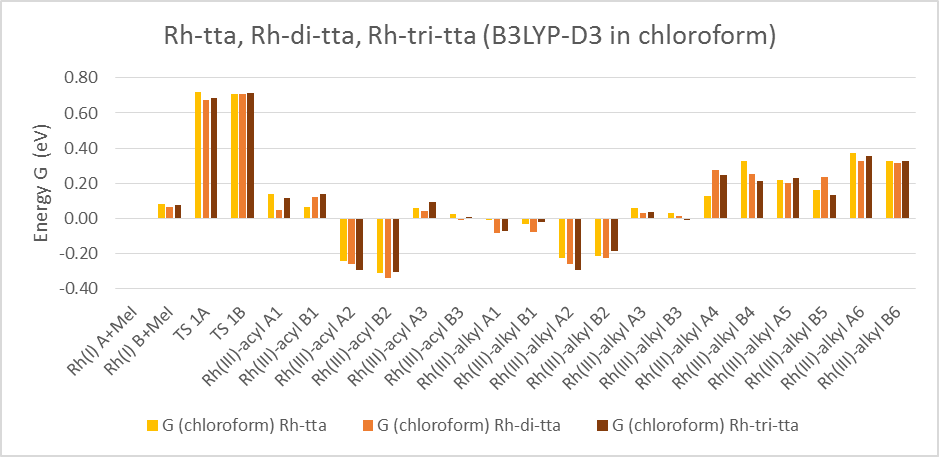


Figure 5. B3LYP-D3 solvent (chloroform) phase relative electronic (E) and free (G) energies of Rh(III)-tta , Rh(III)-di-tta and Rh(III)-tri-tta complexes compared to the lowest energy reactant isomer Rh(I) + Rh(I) + CH_3_I (MeI), illustrating the influence of the amount of thienyl groups on the calculated energy.

Table 1. Electronic (E (eV)) and free energy (G (eV)) data of the indicated reaction products of the [Rh(RCOCHCOCF_3_)(CO)(PPh_3_)] + CH_3_I (MeI), reaction (R = C_4_H_3_S (tta), C_4_H_3_S-C_4_H_2_S (di-tta) and C_4_H_3_S-C_4_H_2_S-C_4_H_2_S (tri-tta)) calculated with B3LYP (with and without dispersion correction) and the indicated phase (gas, chloroform or methanol).

|  | **Rh-tta** | | **Rh-di-tta** | | | | | | | | | | | | **Rh-tri-tta** | |
| --- | --- | --- | --- | --- | --- | --- | --- | --- | --- | --- | --- | --- | --- | --- | --- | --- |
|  | **B3LYP-D3** | | **B3LYP** | | | | | | **B3LYP-D3** | | | | | | **B3LYP-D3** | |
|  | CHCl_3_ | | Gas | | CHCl_3_ | | MeOH | | Gas | | CHCl_3_ | | MeOH | | CHCl_3_ | |
|  | ΔE | ΔG | ΔE | ΔG | ΔE | ΔG | ΔE | ΔG | ΔE | ΔG | ΔE | ΔG | ΔE | ΔG | ΔE | ΔG |
| Rh(I) A + CH_3_I | 0.00 | 0.00 | 0.05 | 0.03 | 0.02 | 0.00 | 0.00 | 0.00 | 0.00 | 0.00 | 0.00 | 0.00 | 0.00 | 0.00 | 0.00 | 0.00 |
| Rh(I) B + CH_3_I | 0.04 | 0.08 | 0.00 | 0.00 | 0.00 | 0.00 | 0.00 | 0.02 | 0.08 | 0.05 | 0.10 | 0.06 | 0.11 | 0.07 | 0.10 | 0.08 |
| TS 1A | 0.13 | 0.72 | - | - | 0.53 | 1.06 | 0.43 | 0.98 | - | - | 0.13 | 0.67 | 0.05 | 0.59 | 0.13 | 0.68 |
| TS 1B | 0.16 | 0.71 | - | - | 0.47 | 0.94 | 0.39 | 0.91 | - | - | 0.21 | 0.71 | 0.14 | 0.60 | 0.21 | 0.72 |
| Rh(III)-acyl A1 | -0.58 | 0.14 | -0.03 | 0.28 | -0.09 | 0.53 | -0.14 | 0.49 | -0.51 | 0.05 | -0.56 | 0.04 | -0.60 | -0.02 | -0.54 | 0.11 |
| Rh(III)-acyl B1 | -0.60 | 0.06 | -0.01 | 0.22 | -0.07 | 0.58 | -0.12 | 0.53 | -0.48 | 0.14 | -0.52 | 0.12 | -0.55 | 0.08 | -0.51 | 0.14 |
| Rh(III)-acyl A2 | -0.99 | -0.24 | -0.43 | 0.20 | -0.47 | 0.16 | -0.54 | 0.12 | -0.89 | -0.27 | -0.92 | -0.26 | -0.77 | -0.10 | -0.93 | -0.29 |
| Rh(III)-acyl B2 | -0.99 | -0.31 | -0.33 | 0.31 | -0.41 | 0.21 | -0.53 | 0.14 | -0.89 | -0.27 | -0.97 | -0.34 | -1.05 | -0.39 | -0.97 | -0.31 |
| Rh(III)-acyl A3 | -0.67 | 0.06 | 0.15 | 0.81 | 0.02 | 0.67 | -0.06 | 0.60 | -0.49 | 0.17 | -0.63 | 0.04 | -0.70 | -0.05 | -0.63 | 0.09 |
| Rh(III)-acyl B3 | -0.71 | 0.02 | 0.03 | 0.20 | -0.06 | 0.62 | -0.12 | 0.57 | -0.58 | 0.09 | -0.67 | -0.01 | -0.72 | -0.02 | -0.68 | 0.01 |
| Rh(III)-alkyl A1 | -0.72 | -0.01 | 0.00 | 0.64 | -0.10 | 0.53 | -0.17 | 0.49 | -0.64 | -0.03 | -0.72 | -0.09 | -0.77 | -0.14 | -0.72 | -0.08 |
| Rh(III)-alkyl B1 | -0.72 | -0.03 | -0.11 | 0.55 | -0.17 | 0.44 | -0.22 | 0.39 | -0.66 | -0.04 | -0.70 | -0.08 | -0.74 | -0.13 | -0.66 | -0.02 |
| Rh(III)-alkyl A2 | -0.91 | -0.23 | -0.19 | 0.44 | -0.24 | 0.39 | -0.28 | 0.35 | -0.81 | -0.21 | -0.86 | -0.26 | -0.90 | -0.31 | -0.93 | -0.29 |
| Rh(III)-alkyl B2 | -0.89 | -0.22 | -0.18 | 0.46 | -0.24 | 0.40 | -0.28 | 0.37 | -0.78 | -0.19 | -0.85 | -0.23 | -0.89 | -0.27 | -0.85 | -0.19 |
| Rh(III)-alkyl A3 | -0.63 | 0.06 | -0.05 | 0.55 | 0.00 | 0.60 | 0.01 | 0.61 | -0.66 | -0.03 | -0.60 | 0.03 | -0.45 | 0.19 | -0.60 | 0.04 |
| Rh(III)-alkyl B3 | -0.63 | 0.03 | -0.03 | 0.56 | 0.01 | 0.58 | 0.01 | 0.59 | -0.65 | -0.04 | -0.59 | 0.01 | -0.58 | 0.02 | -0.59 | -0.01 |
| Rh(III)-alkyl A4 | -0.56 | 0.13 | 0.21 | 0.84 | 0.16 | 0.77 | 0.11 | 0.74 | -0.31 | 0.30 | -0.32 | 0.27 | -0.36 | 0.33 | -0.36 | 0.25 |
| Rh(III)-alkyl B4 | -0.40 | 0.33 | 0.26 | 0.86 | 0.19 | 0.79 | 0.13 | 0.76 | -0.32 | 0.30 | -0.37 | 0.25 | -0.42 | 0.21 | -0.40 | 0.21 |
| Rh(III)-alkyl A5 | -0.47 | 0.22 | 0.19 | 0.78 | 0.22 | 0.78 | 0.21 | 0.81 | -0.46 | 0.16 | -0.43 | 0.20 | -0.43 | 0.20 | -0.43 | 0.23 |
| Rh(III)-alkyl B5 | -0.52 | 0.16 | 0.20 | 0.78 | 0.22 | 0.82 | 0.22 | 0.82 | -0.47 | 0.12 | -0.36 | 0.24 | -0.35 | 0.24 | -0.48 | 0.13 |
| Rh(III)-alkyl A6 | -0.28 | 0.37 | 0.41 | 0.97 | 0.38 | 0.90 | 0.34 | 0.92 | -0.25 | 0.32 | -0.21 | 0.32 | -0.24 | 0.32 | -0.26 | 0.36 |
| Rh(III)-alkyl B6 | -0.32 | 0.33 | 0.25 | 0.85 | 0.26 | 0.86 | 0.25 | 0.87 | -0.30 | 0.28 | -0.27 | 0.31 | -0.26 | 0.32 | -0.24 | 0.33 |

**Experimental Design, Materials and Methods**

Density functional theory (DFT) calculations using the Gaussian 16 package [6], were used to determine the optimized geometry and energy of the spesified molecules. The input coordinates for the compounds were constructed using Chemcraft [7]. The coordinates were spesified in the input files of the DFT calculations. DFT calculations were performed using the hybrid functional B3LYP functional [8] [9] applying the GTO (Gaussian type orbital) triple-ζ basis set 6-311G(d,p) for the lighter atoms (C, H, O, F) and the Lanl2dz basis set [10], that corresponds to the Los Alamos ECP plus DZ, for Rh and I. The optimization is performed using Berny algorithm using GEDIIS [11] as implemented in Gaussian 16. The convergence is reached when the root mean square force, the maximum force, the root mean square displacement and the maximum displacement are within the threshold of 0.00030, 0.00045, 0.0012 and 0.0018 atomic units, respectively. The requested convergence on energy is 1.0D-8 atomic unit. Calculations were done with and without Grimme's D3 dispersion correction [12], in gas and solvent phase, using either chloroform or methanol as solvent. For solvent calculations, the integral equation formalism polarizable continuum model (IEFPCM) of solvation to describe the dielectric continuum medium, was used [13] [14]. Frequency calculations were done on all molecules to ensure true minimum energy (no imaginary frequency) or transtion state structure (one imaginary frequency), and to provide the free energies of the molcules. The free energies were obtained from the output files searching for “Sum of electronic and thermal Free Energies=”. The electronic energies were obtained from the output files at the final optimization step, searching for “SCF Done” from the bottom of the output file.

**Ethics Statement**

This work does not require any ethical statement.

**CRediT author statement**

**Nandisiwe Ghandi Sibongile Mateyise:** DFT calculations, Data curation. **Marrigje M Conradie:** Conceptualization, Supervision, Methodology, Reviewing and Editing. **Jeanet Conradie:** Supervision, Methodology, DFT calculations, Data curation, Writing- Reviewing and Editing.

**Acknowledgments**

This work has received support from the South African National Research Foundation (Grant numbers 129270, 113327, 96111 (JC) and 108960 (MMC)) and the Central Research Fund of the University of the Free State, Bloemfontein, South Africa. The CHPC of South Africa, the High Performance Computing facility of the UFS and the Norwegian Supercomputing Program (UNINETT Sigma2, Grant No. NN9684K) are acknowledged for computer time.

**Declaration of Competing Interest**

The authors declare that they have no known competing financial interests or personal relationships which have or could be perceived to have influenced the work reported in this article.

**References**

[1] N. Ghandi Sibongile Mateyise, J. Conradie, M. Marianne Conradie, Synthesis, Characterization, Electrochemistry, DFT and Kinetic Study of the Oligothiophene-containing Complex [Rh((C_4_H_3_S-C_4_H_2_S)COCHCOCF_3_)(CO)(PPh_3_)], Polyhedron. 115095 (2021). doi:10.1016/j.poly.2021.115095.

[2] M.M. Conradie, J. Conradie, A kinetic study of the oxidative addition of methyl iodide to [Rh((C_4_H_3_S)COCHCOCF_3_)(CO)(PPh_3_)] utilizing UV/vis and IR spectrophotometry and ^1^H, ^19^F and ^31^P NMR spectroscopy. Synthesis of [Rh((C_4_H_3_S)COCHCOCF_3_)(CO)(PPh_3_)(CH_3_)(I)], Inorganica Chim. Acta. 361 (2008) 208–218. doi:10.1016/j.ica.2007.07.010.

[3] M.M. Conradie, J. Conradie, Methyl iodide oxidative addition to [Rh(acac)(CO)(PPh_3_)]: An experimental and theoretical study of the stereochemistry of the products and the reaction mechanism, Dalt. Trans. 40 (2011) 8226–8237. doi:10.1039/c1dt10271k.

[4] M.M. Conradie, J. Conradie, Stereochemistry of the reaction products of the oxidative addition reaction of methyl iodide to [Rh((C_4_H_3_S)COCHCOR)(CO)(PPh_3_)]: A NMR and computational study. R=CF_3_, C_6_H_5_, C_4_H_3_S, Inorganica Chim. Acta. 362 (2009) 519–530. doi:10.1016/j.ica.2008.04.046.

[5] J. Conradie, Density functional theory calculations of Rh-β-diketonato complexes, Dalt. Trans. 44 (2015) 1503–1515. doi:10.1039/C4DT02268H.

[6] M.J. Frisch, G.W. Trucks, H.B. Schlegel, G.E. Scuseria, M.A. Robb, J.R. Cheeseman, G. Scalmani, V. Barone, G.A. Petersson, H. Nakatsuji, X. Li, M. Caricato, A. V. Marenich, J. Bloino, B.G. Janesko, R. Gomperts, B. Mennucci, H.P. Hratchian, J. V. Ortiz, A.F. Izmaylov, J.L. Sonnenberg, D. Williams-Young, F. Ding, F. Lipparini, F. Egidi, J. Goings, B. Peng, A. Petrone, T. Henderson, D. Ranasinghe, V.G. Zakrzewski, J. Gao, N. Rega, G. Zheng, W. Liang, M. Hada, M. Ehara, K. Toyota, R. Fukuda, J. Hasegawa, M. Ishida, T. Nakajima, Y. Honda, O. Kitao, H. Nakai, T. Vreven, K. Throssell, J. Montgomery, J. A., J.E. Peralta, F. Ogliaro, M.J. Bearpark, J.J. Heyd, E.N. Brothers, K.N. Kudin, V.N. Staroverov, T.A. Keith, R. Kobayashi, J. Normand, K. Raghavachari, A.P. Rendell, J.C. Burant, S.S. Iyengar, J. Tomasi, M. Cossi, J.M. Millam, M. Klene, C. Adamo, R. Cammi, J.W. Ochterski, R.L. Martin, K. Morokuma, O. Farkas, J.B. Foresman, D.J. Fox, Gaussian 16, Revision B.01, (2016).

[7] http://www.chemcraftprog.com/, (n.d.) http://www.chemcraftprog.com/.

[8] A.D. Becke, Density-functional exchange-energy approximation with correct asymptotic behavior, Phys. Rev. A. 38 (1988) 3098–3100. doi:10.1103/PhysRevA.38.3098.

[9] C. Lee, W. Yang, R.G. Parr, Development of the Colle-Salvetti correlation-energy formula into a functional of the electron density, Phys. Rev. B. 37 (1988) 785–789. doi:10.1103/PhysRevB.37.785.

[10] P.J. Hay, W.R. Wadt, Ab initio effective core potentials for molecular calculations. Potentials for K to Au including the outermost core orbitale, J. Chem. Phys. 82 (1985) 299–310. doi:10.1063/1.448975.

[11] X. Li, M.J. Frisch, Energy-Represented Direct Inversion in the Iterative Subspace within a Hybrid Geometry Optimization Method, J. Chem. Theory Comput. 2 (2006) 835–839. doi:10.1021/ct050275a.

[12] S. Grimme, J. Antony, S. Ehrlich, H. Krieg, A consistent and accurate ab initio parametrization of density functional dispersion correction (DFT-D) for the 94 elements H-Pu, J. Chem. Phys. 132 (2010) 154104. doi:10.1063/1.3382344.

[13] A. V Marenich, C.J. Cramer, D.G. Truhlar, Universal Solvation Model Based on Solute Electron Density and on a Continuum Model of the Solvent Defined by the Bulk Dielectric Constant and Atomic Surface Tensions, J. Phys. Chem. B. 113 (2009) 6378–6396. doi. 10.1021/jp810292n

[14] R.E. Skyner, J.L. Mcdonagh, C.R. Groom, T. Van Mourik, A review of methods for the calculation of solution free energies and the modelling of systems in solution, Phys. Chem. Chem. Phys. 17 (2015) 6174–6191. doi:10.1039/C5CP00288E.
